# Supplementary material for: The Perme Mobility Index: A new concept to assess mobility level in patients with coronavirus (COVID-19) infection
Source: PLoS One. 2021 Apr 21;16(4):e0250180. doi: 10.1371/journal.pone.0250180 (PMC8059854; doi:10.1371/journal.pone.0250180)
Supplement: S1 Table — Definition of abbreviations: SAPS = simplified acute physiology score; ICU = intensive care unit; CI = confidence interval; GVIF = generalized variance-inflation factors; df = degrees of freedom. *Scores on SAPS III range from 0 to 217, with higher scores indicating more severe illness and higher risk of death. †Charlson comorbidity index range from 0 to 5 for each comorbidity, with score of zero indicating that no comorbidities were found. The higher the score, the more likely the predicted outcome will result in mortality or higher resource use. (DOCX) [file pone.0250180.s001.docx]

| **S1 Table –** Assessment of multicollinearity in the final multivariable model. | | | |
| --- | --- | --- | --- |
|  | **GVIF** | **df** | **GVIF^1/(2*df)^** |
| Age | 2.247795 | 1 | 1.499265 |
| SAPS III score* | 2.546608 | 1 | 1.595809 |
| Charlson comorbidity index^†^ | 1.227772 | 1 | 1.108049 |
| ICU source of admission | 1.099375 | 2 | 1.023968 |
| Use of renal replacement therapy | 1.229394 | 1 | 1.108780 |

*Definition of abbreviations:* SAPS = simplified acute physiology score; ICU = intensive care unit; CI = confidence interval; GVIF = generalized variance-inflation factors; df = degrees of freedom.

*Scores on SAPS III range from 0 to 217, with higher scores indicating more severe illness and higher risk of death.

^†^Charlson comorbidity index range from 0 to 5 for each comorbidity, with score of zero indicating that no comorbidities were found. The higher the score, the more likely the predicted outcome will result in mortality or higher resource use.
