# Supplementary material for: The Perme Mobility Index: A new concept to assess mobility level in patients with coronavirus (COVID-19) infection
Source: PLoS One. 2021 Apr 21;16(4):e0250180. doi: 10.1371/journal.pone.0250180 (PMC8059854; doi:10.1371/journal.pone.0250180)
Supplement: S2 Table — Definition of abbreviations: SAPS: simplified acute physiology score; SOFA = sequential organ failure assessment; ICU = intensive care unit; ECMO = extracorporeal membrane oxygenation; PMI = perme mobility index. Data are median and interquartile range (IQR) values or n (%). Percentages may not total 100 because of rounding. *The body-mass index (BMI) is calculated by weight in kilograms divided by the square of the height in meters. †Scores on SAPS III range from 0 to 217, with higher scores indicating more severe illness and higher risk of death. ‡SOFA scores range from 0 to 4 for each organ system, with higher aggregate scores indicating more severe organ dysfunction. §Charlson comorbidity index range from 0 to 5 for each comorbidity, with score of zero indicating that no comorbidities were found. The higher the score, the more likely the predicted outcome will result in mortality or higher resource use. ||Other–includes other hospitals, ambulatory, procedure rooms, CT scan room, and other hospital units. ¶Organ support during ICU stay. **Perme ICU mobility score range from 0 to 32, with higher scores indicating better mobility level.††At ICU discharge or death. (DOCX) [file pone.0250180.s002.docx]

| **S2 Table** – Baseline characteristics of patients with or without missing in Perme Score. | | | |  |
| --- | --- | --- | --- | --- |
|  | **Missing in Perme**  **(*n* = 64)** | **No Missing in Perme**  **(*n* = 136)** | ***P* Value** |  |
| Age, years | 57.5 (48.8–69.8) | 69.0 (53.0–82.0) | 0.032 |  |
| Male gender – no. (%) | 45 (70.3) | 78 (57.4) | 0.088 |  |
| Body mass index* | 27.8 (25.4–31.8) | 28.3 (24.9–32.0) | 0.875 |  |
| Severity of illness |  |  |  |  |
| SAPS III score^†^ | 44.0 (42.0–51.2) | 53.0 (45.0–60.0) | < 0.001 |  |
| SOFA^‡^ | 1.0 (0.0–2.0) | 4.0 (2.0–7.0) | < 0.001 |  |
| Charlson comorbidity index^§^ | 0.0 (0.0–2.0) | 1.0 (0.0–2.0) | 0.139 |  |
| Modified frailty index | 1.0 (0.0–2.0) | 1.0 (0.0–3.0) | 0.055 |  |
| Score | 0.1 (0.0–0.2) | 0.1 (0.0–0.2) | 0.057 |  |
| Clinical frailty – no. (%) | 5 (7.8) | 12 (8.8) | 1.000 |  |
| ICU source of admission – no. (%) |  |  | 0.471 |  |
| Emergency department | 31 (48.4) | 65 (47.8) |  |  |
| Ward | 25 (39.1) | 54 (39.7) |  |  |
| Step down unit | 6 (9.4) | 5 (3.7) |  |  |
| Other^\|\|^ | 2 (3.1) | 12 (8.8) |  |  |
| Organ support – no. (%) |  |  |  |  |
| Non-invasive ventilation | 26 (40.6) | 105 (77.2) | < 0.001 |  |
| Invasive ventilation | 7 (10.9) | 82 (60.3) | < 0.001 |  |
| Endotracheal tube | 6 / 7 (85.7) | 71 / 82 (86.6) |  |  |
| Tracheostomy | 1 / 7 (14.3) | 11 / 82 (13.4) | 1.000 |  |
| Renal replacement therapy | 3 (4.7) | 31 (22.8) | 0.001 |  |
| ECMO | 1 (1.6) | 0 (0.0) | 0.320 |  |
| Perme ICU Mobility Score^**^ |  |  |  |  |
| At admission | - | 7.0 (0.0–16.0) | - |  |
| At final follow-up^††^ | - | 20.0 (7.0–28.0) | - |  |
| Difference | - | 4.5 (0.0–16.2) | - |  |
| Perme mobility index | - | 0.6 (0.0–2.0) | - |  |
| *Definition of abbreviations:* SAPS: simplified acute physiology score; SOFA = sequential organ failure assessment; ICU = intensive care unit; ECMO = extracorporeal membrane oxygenation; PMI = perme mobility index.  Data are median and interquartile range (IQR) values or n (%). Percentages may not total 100 because of rounding.  *The body-mass index (BMI) is calculated by weight in kilograms divided by the square of the height in meters.  ^†^Scores on SAPS III range from 0 to 217, with higher scores indicating more severe illness and higher risk of death.  ^‡^SOFA scores range from 0 to 4 for each organ system, with higher aggregate scores indicating more severe organ dysfunction.  ^§^Charlson comorbidity index range from 0 to 5 for each comorbidity, with score of zero indicating that no comorbidities were found. The higher the score, the more likely the predicted outcome will result in mortality or higher resource use.  ^\|\|^Other – includes other hospitals, ambulatory, procedure rooms, CT scan room, and other hospital units.  ^¶^Organ support during ICU stay.  **Perme ICU mobility score range from 0 to 32, with higher scores indicating better mobility level.  ^††^At ICU discharge or death. | | | | |
