# Supplementary material for: The Perme Mobility Index: A new concept to assess mobility level in patients with coronavirus (COVID-19) infection
Source: PLoS One. 2021 Apr 21;16(4):e0250180. doi: 10.1371/journal.pone.0250180 (PMC8059854; doi:10.1371/journal.pone.0250180)
Supplement: S3 Table — Definition of abbreviations: ICU = intensive care unit. Data are median and interquartile range (IQR) values or n (%). Percentages may not total 100 because of rounding. (DOCX) [file pone.0250180.s003.docx]

| **S3 Table –** Clinical outcomes in patients with or without missing in Perme Score. | | | |
| --- | --- | --- | --- |
|  | **Missing in Perme**  **(*n* = 64)** | **No Missing in Perme**  **(*n* = 136)** | ***P* Value** |
| Duration of ventilation (days) | 5.0 (4.0–23.5) | 11.0 (7.0–18.0) | 0.450 |
| In survivors (days) | 5.0 (4.5–24.0) | 10.0 (6.0–15.0) | 0.666 |
| ICU length of stay (days) | 3.0 (2.0–5.0) | 12.0 (7.0–23.2) | < 0.001 |
| In survivors (days) | 3.0 (2.0–4.0) | 11.0 (6.2–21.0) | < 0.001 |
| Hospital length of stay (days) | 8.5 (5.0–12.2) | 19.5 (12.2–35.0) | < 0.001 |
| In survivors (days) | 8.0 (5.2–12.0) | 19.5 (12.0–35.2) | < 0.001 |
| ICU mortality – no. (%) | 7 (10.9) | 22 (16.2) | 0.393 |
| Hospital mortality – no. (%) | 10 (15.6) | 22 (16.2) | 0.999 |
| *Definition of abbreviations:* ICU = intensive care unit.  Data are median and interquartile range (IQR) values or *n* (%). Percentages may not total 100 because of rounding. | | | |
