# Supplementary material for: The Perme Mobility Index: A new concept to assess mobility level in patients with coronavirus (COVID-19) infection
Source: PLoS One. 2021 Apr 21;16(4):e0250180. doi: 10.1371/journal.pone.0250180 (PMC8059854; doi:10.1371/journal.pone.0250180)
Supplement: Attachment — Submitted filename: Manuscript Number PONE-D-21-01443.pdf [file pone.0250180.s005.pdf]

I appreciate the invitation to review the article. This is an interesting article, but it has some aspects that can improve. I respectfully attach my comments.

Authors might revise grammatic aspects of the manuscript.

Title

The title might include the index used to identify the mobility level. Avoid the use of many articles in the title.

Introduction

It clearly justifies the interest of the chosen topic in the studied population and highlights the importance of the study. The authors might think of emphasize in the introduction the new concept proposed regarding the Index.

Methods

The study design is a retrospective cohort study, given that the information was taken from electronic medical records, the authors should revise the wording of the inclusion and exclusion criteria to avoid confusion regarding the temporality of the study. For example, instead of “Patients admitted to the ICU with diagnosis of COVID-19…”, the authors might consider change it to “the clinical records of patients admitted to the ICU with a confirmed diagnosis of COVID 19…”

Authors must describe the process of construction/validation of the new index presented in this study “The Perme Mobility Index (PMI)”. This is a very important contribution and given that it has never been done, a validation process is necessary.

Discussion

Authors also, might consider using the minimum detectable difference of the Perme Score that already published in the discussion section.
